# Assessing the potential for fall armyworm exchanges between the two American continents across the Mexico-Central America land bridge

**DOI:** 10.1371/journal.pone.0308501

**Published:** 2025-03-03

**Authors:** Rodney N. Nagoshi, Edi A. Malo, Samuel Cruz-Esteban, Ninfa M. Rosas-García, Verónica Herrera-Mayorga, Robert L. Meagher

**Affiliations:** 1 Center for Medical, Agricultural and Veterinary Entomology, United States Department of Agriculture-Agricultural Research Service, Gainesville, Florida, United States of America; 2 Departamento de Ecología de Artrópodos y Manejo de plagas, El Colegio de la Frontera Sur (ECOSUR), Tapachula, Chiapas, México; 3 Laboratorio de Biotecnología Ambiental del Centro de Biotecnología Genómica del Instituto Politécnico Nacional, Reynosa, Tamaulipas, México; 4 Unidad Académica Multidisciplinaria Mante, Universidad Autónoma de Tamaulipas, El Mante, Tamaulipas, Mexico; University of Saskatchewan College of Agriculture and Bioresources, CANADA

## Abstract

The fall armyworm is a major agricultural pest of corn and multiple other crops. A native of the Western Hemisphere it is now a global concern after its discovery in Africa in 2016 and subsequent infestations in Asia and Australia. A defining characteristic of fall armyworm is its capacity for long distance migration, first documented for populations in North America and assumed to occur elsewhere. This trait increases the risk that phenotypes harmful to agriculture, such as pesticide resistance, that arise in one location can rapidly disseminate to even geographically distant populations. This study examines the extent to which the Mexico-Central America land bridge serves as a pathway for gene flow between the fall armyworm from the two American continents. The work builds on previous surveys in Mexico that suggest possible geographical or meteorological barriers limiting mass population movements. Fall armyworm collections from Mexico were analyzed for patterns of genetic variation and these were correlated with modeling of climate suitability, the locations of major corn production, and wind-dependent dispersal projections. Significant but transient genetic structure was found in Mexico consistent with climate suitability and air transport projections that support localized dispersal behavior rather than long-distance movements. The results indicate the movement of fall armyworm through Mexico does not involve migratory movements of mass populations as observed in the United States. This suggests that the exchange of fall armyworm between the two Americas across the Central America land bridge is limited in scope and frequency.

## Introduction

Invasive pest insects are a growing problem as climate change and urbanization destabilize ecosystems and international trade facilitates the introduction of species into new locations. A related concern is that this increased mobility enhances the gene flow between geographically distant populations, thereby facilitating the dispersion of traits harmful to agriculture across the host range. The risk is magnified in species capable of long-distance flight as this allows transient expansion even into regions that only support viability for a portion of the year. A model for this characteristic is the noctuid moth *Spodoptera frugiperda* (JE Smith) (Lepidoptera: Noctuidae), commonly known as fall armyworm (FAW). Although a semi-tropical species incapable of surviving periods of prolonged freezing temperatures, FAW infestations are found in most locations capable of corn production, even in those with severe winters [1]. In North America, this occurs through annual mass migrations from locations with moderate winter conditions (southern Texas and Florida) to agricultural areas as far north as Canada, a distance spanning thousands of kilometers across latitudes that only allow FAW development during the spring and summer[2,3].

A model of long-distance FAW population movements in North America was developed based upon seasonal wind patterns, the timing and extent of corn plantings, and the temperature-dependent rate of FAW development [3]. The projected dispersion patterns closely corresponded to the actual migration pathways as mapped by a combination of field collections and genetic haplotypes [4]. This indicated that the observed FAW northward advance is primarily dictated by favorable wind conditions and the availability of sufficient host plants to support dense populations. The migration occurs through a progression of nocturnal flights exhibited over multiple generations beginning in the early spring and extending into the fall season.

This documented capacity in North America to move long distances is consistent with whole-genome polymorphism analyses indicating a single interbreeding, panmictic population occupying the Western Hemisphere [5,6]. However, more detailed genetic surveys have identified two instances of location-dependent genetic structure that have persisted over multiple years of surveys. The first is the division of FAW into two morphologically indistinguishable groups historically called “host strains” based on the biased association of molecular markers in FAW found in different habitats and plant hosts [7]. The corn-strain is preferentially found infesting corn, sorghum, and cotton while the rice-strain predominates in habitats dominated by pasture and forage grasses, millet, and alfalfa. The rice-strain was initially identified using FAW collected on rice, however subsequent studies indicate that rice is probably a secondary host with a variable (though still biased) strain association [8]. To avoid misleading implications of host specificity, we have taken to referring to the corn-strain and rice-strain as C-strain and R-strain, respectively.

The second instance of population structure is observed within the C-strain group. A subset of polymorphisms within the 3’ portion of the mitochondrial Cytochrome Oxidase Subunit I (*COI*) gene (COIB) define four haplotype classes (designated CSh1-4) that show reproducible regional differences in their proportions [8]. While all four CSh haplotypes are found in both Americas, their relative frequencies in the FAW overwintering in Florida and the Caribbean differ from populations found in the rest of the hemisphere in a pattern that has persisted over multiple years of surveys [9–11].

The seeming contradiction between evidence for a single interbreeding population and observations of genetically distinct groups can be reconciled if there is sufficient gene flow to broadly distribute most genetic markers across the hemisphere but not enough to homogenize the relative frequencies of certain haplotypes. Gene flow limitations between the host strains appears to be maintained by a combination of differential host plant use and partial reproductive incompatibility but these still allow some mating between strains in both laboratory and wild populations [8,12,13]. Furthermore, there is evidence that host strain differentiation may be primarily, if not solely, driven by genes on the *Z*-chromosome [8,14]. If correct, then strain fidelity can be maintained even with complete mixing of the autosomes. Consequently, the effectiveness of whole genome SNP approaches to differentiate the strains may depend on the representation of the *Z*-chromosome in the analysis. In contrast, no phenotypic differences or reproductive incompatibilities have been found for the *COI* CSh-defined populations. The restricted gene flow between these groups appears to be due entirely to their geographical separation with their genetic differentiation limited (so far) to differences in mitochondrial haplotype frequencies.

The C-strain is found in both Americas and FAW overwintering in southern Texas exhibits the same CSh-haplotype profile as those from South America [9], leaving open the possibility of a genetically homogeneous population extending between these geographically distant locations. One way this could occur by natural mechanisms is if there is substantial migration and gene flow across the Mexico-Central America land corridor. Under this scenario, Mexico would have a critical role in U.S. food security as it would serve as a conduit for individuals or alleles from South America entering the overwintering population that is the source of FAW infestations for much of North America.

However, initial genetic analyses of FAW from Mexico, which lies between Texas and Central America, found evidence for only limited interactions within Mexico as well as between Mexico and Texas [15]. If correct, it suggests FAW movements in Mexico occur primarily through local dispersal that is too limited to genetically homogenize the Mexican FAW populations or to significantly impact the haplotype profiles of the Texas populations [16]. This argues against substantial and consistent gene flow between the two American continents and describes a very different migratory behavior than the mass population movements observed with North American FAW. A more detailed investigation of conditions in Mexico could provide new insights into the environmental factors that dictate FAW population movements and could have more general implications for invasive pest monitoring to the extent that FAW serves as a model for other migratory moth species.

There are two objectives to this study. The first is to use additional sampling from Mexico to assess the stability and persistence of the genetic structure previously observed. This will involve genetic comparisons using the previously used CSh markers and the addition of a highly variable intron segment from the *Z*-linked Triosephosphate isomerase (*Tpi*) gene. The second is to examine meteorological and physical factors that might explain the observed haplotype distribution patterns. The results will be relevant to the question of whether a consistent exchange of FAW populations between the two Americas can occur by natural migration across Central America. This would have important implications on the hemispheric spread of pesticide resistant traits in FAW and more generally on the likelihood of Mexico being an entry point for invasive moth species from South America.

## Materials and methods

### Source of specimens

Sample collections were primarily done in locations with substantial corn production as these can support high density FAW populations that simplifies sample collection and the logistics of field surveys. Collections from corn-dominated habitats are predominantly of the C-strain with R-strain captures often infrequent and sporadic. For this reason, this study is limited to C-strain specimens as defined by genetic markers.

The FAW specimens analyzed in this study are described in Table 1 with the abbreviations denoting collection location and year. The collections from Texas (TX), Nebraska (NE), and Pennsylvania (PA) were adult males obtained by pheromone trapping using plastic Universal moth traps (Unitraps) baited with a 2-component mix designated “Fall Armyworm-PSU” lure (Scentry Biologicals, Inc., Billings, MT) and containing insecticide strips (Hercon Environmental Co., Emigsville, PA). The first set of specimens from Mexico were described in a previous study [15] and included colonies derived from larvae collected from cornfields near the cities of Guasave, Sinaloa (Si13), Durango, Durango (Du13), Mante, Tamaulipas (Ta13), and Tapachula, Chiapas (Ch13). These had gone through < 7 generations in culture. Field collections were obtained in 2014 from Durango (Du14) and Tamaulipas (Ta14) at approximately the same locations as the sources of the colonies [15]. Additional specimens from Mexico were obtained by field collections of larvae that were raised to adulthood in the laboratory and allowed to mate to produce colonies. After laying eggs, the field collected parents were used for the genetic analysis representing the states of Yucatan (Yu15), Jalisco (Ja15), Michoacán (Mi14), Morelos (Mo15), and Campeche (Ca15) [17], while a subset of the F1 generation was used for the Chiapas analyses (Ch16). The specimens were mailed to CMAVE, Gainesville, Florida for DNA preparation. All collections were derived from larvae picked directly from corn or adults obtained by sweep netting or pheromone trapping in cornfields.

**Table 1:** Source information for collections.

Abbreviation	County, State	Country	Year	Type^1^	Collector/Reference
Br04	Brazos, TX	USA	2004	F	[18]
TG06	Tom Green, TX	USA	2006	F	[18]
Hi06-8	Hidalgo, TX	USA	2006-8	F	[18]
Hi11	Hidalgo, TX	USA	2011	F	[15]
Nu11	Nueces, TX	USA	2011	F	[19]
Nu12,15	Nueces, TX	USA	2012, 2015	F	[16]
Lu21	Lubbock, TX	USA	2021	F	[16]
NE15	Scotts Bluff, NE	USA	2015	F	[15]
PA15	Erie, PA	USA	2015	F	[20]
Ch13	Chiapas	Mexico	2013	C	[15]
Du13	Durango	Mexico	2013	C	[15]
Si13	Sinaloa	Mexico	2013	C	[15]
Ta13	Tamaulipas	Mexico	2013	C	[15]
Du14	Durango	Mexico	2014	F	[15]
Ta14	Tamaulipas	Mexico	2014	F	[15]
Du16, 17	Durango	Mexico	2016-2017	F	Ninfa M. Rosas García
Ch16	Chiapas	Mexico	2016	F	Edi A. Malo Rivera
Yu15	Yucatan	Mexico	2015	F	Edi A. Malo Rivera
Ja15	Jalisco	Mexico	2015	F	Edi A. Malo Rivera
Mi14	Michoacán	Mexico	2014	F	Edi A. Malo Rivera
Mo15	Morelos	Mexico	2015	F	Edi A. Malo Rivera
Ca15	Campeche	Mexico	2015	F	Edi A. Malo Rivera

^1^C: colony, F: field collection

### DNA sequence analysis

Nuclear and mitochondrial DNA were isolated from single specimens by homogenization in a 5-ml Dounce homogenizer (Thermo Fisher Scientific, Waltham, Massachusetts, USA) in 1 ml of phosphate buffered saline (PBS, 20 mM sodium phosphate, 150 mM NaCl, pH 8.0). The homogenate was transferred to a 2-ml microcentrifuge tube and pelleted by centrifugation at 6000 g for 5 minutes at room temperature. The pellet was resuspended in 400 µl Genomic Lysis buffer (Zymo Research, Orange, California, USA) and incubated at 55°C in a dry bead bath for at least three hours. Debris was removed by centrifugation at 10,000 rpm for 5 minutes. The supernatant was transferred to a Zymo-Spin III column (Zymo Research, Orange, California, USA) and processed according to manufacturer’s instructions. The collections in Table 1 from earlier published studies were kept in storage as DNA at -20°C. Additional sequence analyses of these specimens were performed as needed.

The relevant segments from the *COI* and *Tpi* genes were amplified by polymerase chain reaction (PCR) amplification in a 30-µl reaction mix containing 3 µl of 10X manufacturer’s reaction buffer, 1 µl 10mM dNTP, 0.5 µl 20- µ M primer mix, 1 µl DNA template (between 0.05-0.5 µg), 0.5 units Taq DNA polymerase (New England Biolabs, Beverly, Massachusetts) with the remaining volume water. The thermocycling program was 94°C (1 min), followed by 30 cycles of 92°C (30 s), 56°C (45 s), 72°C (45 s), and a final segment of 72°C for 3 min. Primers used for the PCR amplification of COIB are c891F (5’-TACACGAGCATATTTTACATC-3’) and c1472R (5’-GCTGGTGGTAAATTTTGATATC-3’). Primers for the *Tpi* segment used for strain identification and that includes the TpiI4a200 intron fragment are t412F (5’-CCGGACTGAAGGTTATCGCTTG-3’) and t1140R (5’-GCGGAAGCATTCGCTGACAACC-3’).

The PCR products were separated by agarose gel electrophoresis and purified using the Zymoclean Gel DNA Recovery Kit (Zymo Research, Orange, California). The isolated fragments were directly analyzed by DNA sequencing performed by either Azenta Life Sciences (Chelmsford, Massachusetts) or Eurofins Genomics (Louisville, Kentucky). All primers were synthesized by Integrated DNA Technologies (Coralville, Iowa).

#### Characterization of the COIB CSh genetic markers.

The CSh haplotypes are derived from two polymorphic sites (mCOI1164D and mCOI1287R) found in the 3’ half (COIB) of the mitochondrial *COI* gene. The mCOI1164D site contains either an A, G, or T while site mCOI1287R varies between A or G. The configuration T_1164_A_1287_ is diagnostic of the R-strain, while four variants define the C-strain, A_1164_A_1287_ (CSh1), A_1164_G_1287_ (CSh2), G_1164_A_1287_ (Csh3), G_1164_G_1287_ (CSh4) [10,18]. The CSh3 haplotype was not found in this study.

#### Characterization of the TpiI4a200 genetic marker.

**Fig 1:**
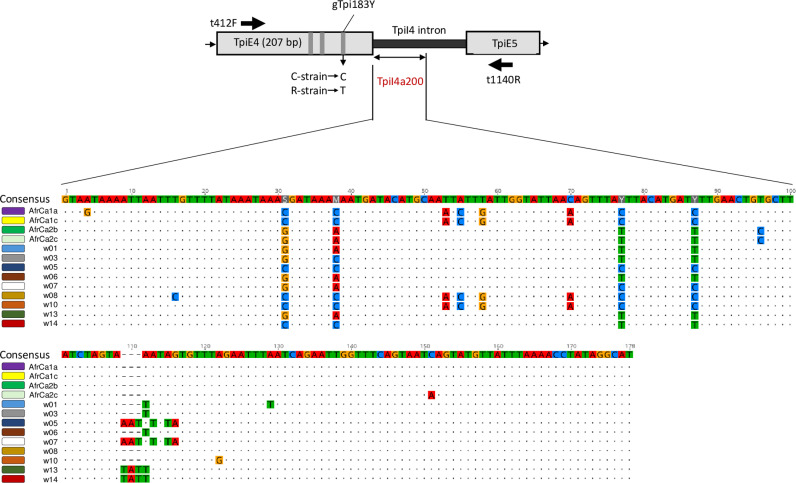
Portion of the *Tpi* gene containing the TpiI4a200 segment of the TpiI4 intron. Grey vertical bars identify strain-specific SNPs in exon with gTpi183Y indicated. The sequences of the thirteen TpiI4a200 variants identified in this study are shown with dots indicating identity with the consensus. The approximate locations and orientation of the t412F and t1140R primers used to isolate TpiI4a200 are indicated by horizontal arrows. The diagram is not drawn to scale.

The fourth exon of the *Tpi* gene contains three polymorphic sites (gTpi165Y, gTpi168Y, and gTpi183Y) that can serve as host strain markers, with gTpi183Y showing the most consistent correspondence with the host stain phenotype [14,21]. Only specimens expressing the C-strain gTpi183Y variant were further analyzed in this study. The intron following this exon, TpiI4, displays high sequence variation and is of variable size because of frequent indels [22]. This sequence complexity can be useful for taxonomic and population studies but is also problematic in a diploid organism where heterozygosity is common. In this case, *Tpi* is Z-linked and so present in two copies in males. Because we directly sequence the PCR product amplified from the nuclear genome, heterozygosity for polymorphisms within the region of interest will produce ambiguous sequence data in the form of overlapping chromatogram curves. Such heterozygous specimens were not included in subsequent *Tpi* sequence analysis. To reduce the frequency of heterozygosity in our region of interest and thereby increase the number of useable sequences, we limited the analysis to the first 60% of the intron, designated TpiI4a200, that approximates 200-bp (Fig 1).

The TpiI4a200 segment was sequenced for each specimen. Distinct sequences were identified as haplotypes and were compared by phylogenetic analysis to 173 TpiI4a200 sequences from Argentina, Brazil, and Florida that had previously been characterized for their strain identity [14]. Phylogenetic trees were produced with Geneious Prime 2021.1.1 software[23] using the Maximum Likelihood [24] method. All analyses underwent bootstrap testing (100 replicates) with the optimal tree shown and drawn to scale. The evolutionary distances were computed using the Maximum Composite Likelihood method [25].

### Climate suitability analysis

CLIMEX is a dynamic simulation model that estimates the potential geographical distribution and relative abundance of a species according to climatic factors such as temperature and moisture [26]. CLIMEX projections for FAW has been performed by multiple groups (e.g., [2,27–29]), and while there were a few variations in the biological parameters used between studies these generally generated only modest differences in the distribution maps. We performed a CLIMEX suitability analysis using the parameters of Timilsena et al, 2022 to generate a more detailed projection of Mexico ([2], Table 2). Climate information was imported from Climond (www.climond.org)[30], using historical data from 1961-1990 at a resolution of 10’. The Compare Locations (1 species) function in the CLIMEX program was used with the Grid Data simulation file. Mexico is considered an arid to semi-arid country with substantial areas of cropland receiving irrigation [31]. To account for the potential additional risk of FAW infestations posed by corn supported by irrigation, a parameter of 2.5 mm day^ − 1^ as top-up irrigation throughout the year when the weekly rainfall was less than 25 mm was added (Irrigation scenario I in [2]).

**Table 2:** CLIMEX parameter values used for modelling fall armyworm (from [2]).

Parameter	Description	Value
Moisture		
SM0	Lower soil moisture threshold	0.15
SM1	Lower optimal soil moisture	0.8
SM2	Upper optimal soil moisture	1.5
SM3	Upper soil moisture threshold	2.0
.5Temperature		
DV0	Lower temperature threshold	12°C
DV1	Lower optimal temperature	25°C
DV2	Upper optimal temperature	30°C
DV3	Upper temperature threshold	36°C
Cold Stress		
TTCS	Cold stress temperature threshold	98°C
THCS	Cold stress accumulation rate	−0.005 week^−1^
Heat Stress		
TTHS	Heat stress temperature threshold	39°C
THHS	Heat stress accumulation rate	0.0025 week^−1^
Dry Stress		
SMDS	Soil moisture dry stress threshold	0.1
HDS	Dry stress accumulation rate	−0.005 week^−1^
Wet Stress		
SMWS	Soil moisture wet stress threshold	3
HWS	Wet stress accumulation rate	0.002 week^−1^
Minimum degree-day sum needed to complete a generation		
PDD	Degree-Days per generation	400°C
	Irrigation	2.5 mm/day

The Growth Index (GI) and Ecoclimatic Index (EI) are outputs from CLIMEX. They are presented on a 0–100 scale, where 0 is classified as not suitable and 100 as 100% suitability (as would occur in an incubator) for part of the year (GI) or all year (EI). The map was set at 0.2 diameter circles, showing zeros with 10 legend items. The Cold Stress projections are presented on a 0-1000 scale, with 1000 representing maximum stress.

### HYSPLIT air trajectory projections

Air transport trajectories for select locations were estimated using the Hybrid Single Particle Lagrangian Integrated Trajectory Model at the Air Resources Laboratory (ARL) READY web site run by NOAA (http://ready.arl.noaa.gov/HYSPLIT.php)[32]. Projections were made using wind vectors whose direction and velocity were averaged for the 30-day periods of March 1-30, 2020 and September 1-30, 2020. Fall armyworm migrates nocturnally so the duration of continuous flight was limited to a 12-hour period beginning at dusk, with a starting altitude of 500 m above ground level (AGL) and a maximum altitude of 1500 m AGL. The pathways of the projections from each location were averaged and displayed as a frequency distribution with percentages reflecting the proportion of trajectories entering a given grid. While FAW can in principle fly on multiple consecutive nights to move very long distances, this is presumed to depend on landing areas that allow sufficient rest, rehydration, and nourishment during the day. This information was not available for the Mexico locations. To simplify the analysis, projections are shown for only a single night’s flight.

## Results

### Distribution of the *COI* haplotypes

**Fig 2:**
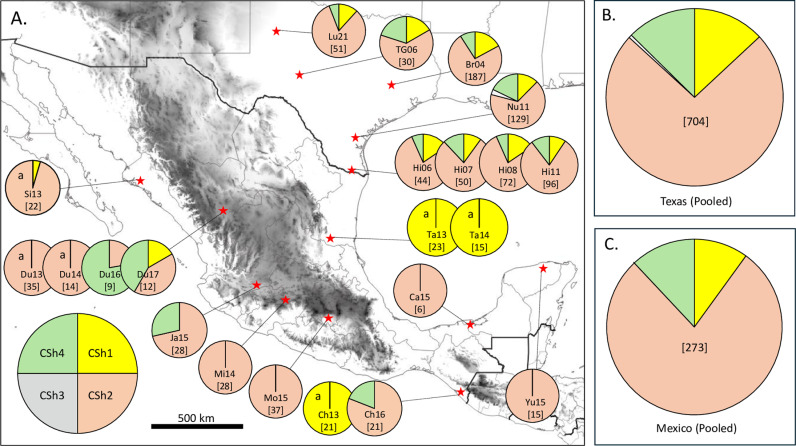
Distribution of the COIB CSh haplotypes in collections from Mexico and Texas. A, locations of the collection sites (red stars with abbreviations as in Table 1) on a map showing elevation (elevation increases with shading, with black indicating > 5000 meters above ground level). Pie charts show relative proportions of the CSh haplotypes from each collection. In brackets under the year are the number of specimens analyzed. Pie charts designated with an “a” are from an earlier study [15]. B, CSh haplotype profile from the pooled Texas collections (n =  704). C, CSh haplotype profile from the pooled Mexico collections (n =  273).

The initial evidence for FAW population structure in Mexico came from the analysis of COIB CSh variants (pie charts labeled “a” in Fig 2A, [15]). The CSh patterns of populations on the western coast, Sinaloa (Si13) and Durango (Du13, Du14), were predominantly or entirely CSh2. In comparison, the populations tested from Chiapas (Ch13) to the south and Tamaulipas (Ta13, Ta14) on the western coast were entirely CSh1, a minor haplotype in other Western Hemisphere collections [9].

Additional specimens were obtained for Durango (Du16, Du17) and Chiapas (Ch16), allowing an examination of the stability of the haplotype profiles at these locations over time. The Durango populations showed a substantial change in haplotype composition from 2013-2014 to 2016-2017 primarily due to the addition of CSh4. The CSh4 haplotype was not observed in our initial survey of Mexico [15] and is a minority component (approximately 12%) of Texas populations (Fig 2B), though it is the predominant haplotype in populations overwintering in Florida [33]. The haplotype profile in Chiapas also changed dramatically from being entirely CSh1 in 2013 to a combination of CSh2 and CSh4 in the 2016 collection, with CSh2 the majority haplotype.

Specimens were also obtained from five other Mexican states, Campeche (Ca15), Jalisco (Ja15), Michoacan (Mi14), Morelos (Mo15), and Yucatan (Yu15). In all but one collection, the specimens were all CSh2 (Fig 2A). The exception was the 2015 collection from Jalisco (Ja15), which was majority CSh2 but with a sizeable number of CSh4 as well.

As a comparison, specimens were obtained from five counties in Texas (Brazos, Hidalgo, Lubbock, Nueces, and Tom Green), which are believed to represent a single interbreeding population that is the primary migratory source for infestations in central and portions of eastern North America [33]. Hidalgo County is probably a major overwintering location for FAW, with the existence of a large permanent population supported by the similarity in the haplotype profile from 2006 to 2011 (Hi06, Hi07, Hi08, Hi11, Fig 2A). This pattern of a majority CSh2 composition with a near equal number of CSh1 and CSh4 was also found in the collections from the other TX counties. The very infrequent CSh3 haplotype was only found in a single specimen in the Nuesces Nu11 collection.

Given the consistency of the Texas CSh haplotype frequencies over time and space, it is not surprising that the pooled Texas CSh profile resembled that found for each Texas collection (Fig 2B). What was unexpected was that despite the considerable variation observed between the different Mexican collections, the pooled Mexico CSh profile is very similar to the Texas profile (Fig 2C).

### Distribution of the *Tpi* intron haplotypes

The TpiI4a200 segment is from the 5’ portion of the fourth intron in the *Tpi* coding sequence (Fig 1). It empirically shows a higher frequency of genetic variation than the mitochondrial *COI* and can thereby serve as a *Z-*chromosome genetic marker with potentially greater resolution for population comparisons [14]. The TpiI4a200 DNA sequence was obtained from 305 specimens, which were comprised of 129 specimens from three counties in Texas, 134 from seven states in Mexico, and 42 from two migratory destinations of the Texas population (Scottsbluff County, Nebraska and Erie County, Pennsylvania). A total of 13 TpiI4a200 haplotypes were found that were initially identified as being of the C-strain using the *Tpi* exon marker, gTpi183Y (Fig 1).

**Fig 3:**
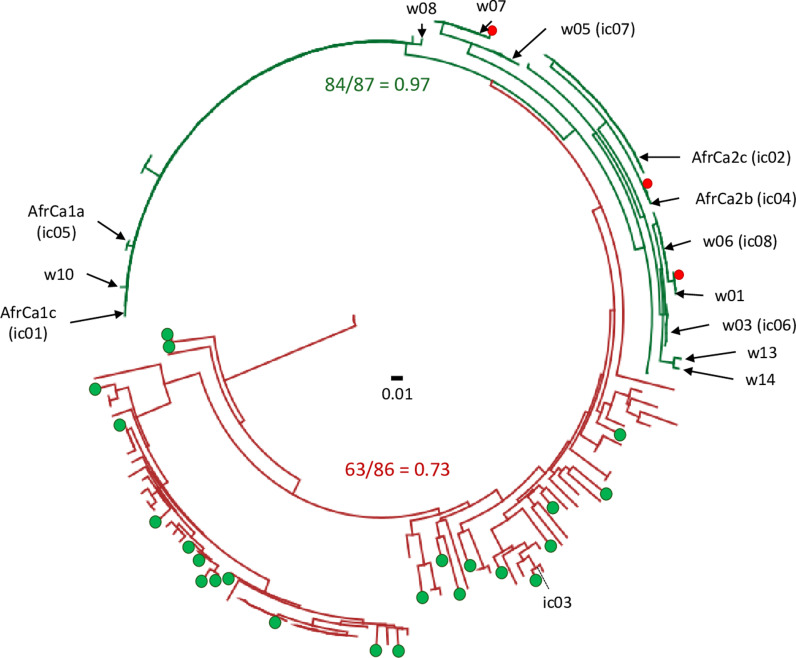
Maximum-likelihood phylogeny of TpiI4a200 from 173 larval specimens from Argentina, Brazil, and Florida ([ **14]) plus the 13 haplotypes from Texas and Mexico.** Red and green lines indicate phylogenetic groups identified as R-strain and C-strain, respectively. Colored circles identify exceptions where the host plant origin of the specimen disagrees with the strain identity. Numbers in red and green show the frequency of agreement between host plant and strain identity. The placement of the 13 Texas and Mexico haplotypes from this study are indicated by arrows. Shown in parentheses are TpiI4a200 haplotypes described in an earlier study using a different nomenclature (ic01-8, [16]). One haplotype, ic03, was initially identified as C-strain by the gTpi183Y marker but groups with the R-strain in the phylogenetic tree.

Strain identification was confirmed by phylogenetic analysis against an existing sequence database. An earlier study demonstrated that a phylogeny constructed from TpiI4a200 sequences identify groupings that correspond to the two FAW host strains [14]. This is illustrated in Fig 3 where a TpiI4a200 phylogeny was constructed from 173 larval specimens collected from either C-strain or R-strain associated host plants from locations in Florida (USA), Argentina, and Brazil. The green lines identify the C-strain grouping where 97% of the individual were from a C-strain host. In the remaining R-strain grouping (red lines) 73% were from R-strain hosts. All 13 of the TpiI4a200 haplotypes identified as C-strain by the *Tpi* exon marker were found in the C-strain phylogenetic grouping (Fig 3).

**Fig 4:**
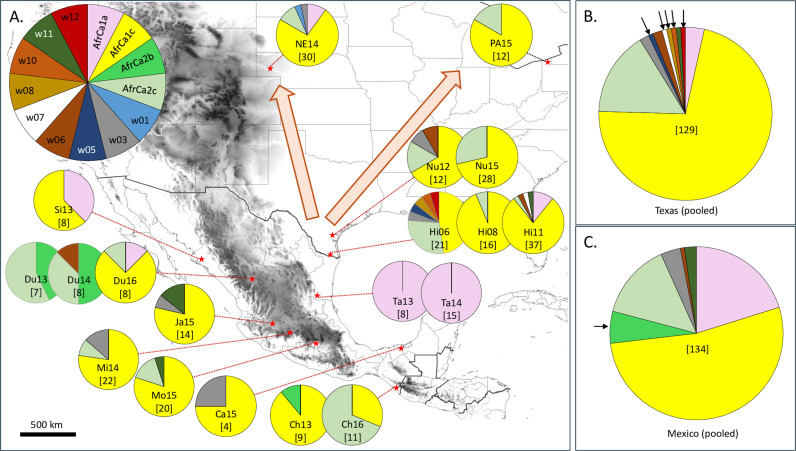
Distribution of the TpiI4a200 haplotypes in collections from Mexico and Texas.

We next analyzed the distribution of the TpiI4a200 haplotypes at different locations. The collection of TpiI4a200 sequences from this study were organized by haplotype for each location and the relative frequencies calculated to produce a haplotype profile, which is illustrated in a pie chart (Fig 4). The five Texas TpiI4a200 haplotype profiles from two locations and five years were similar in that the AfrCa1c haplotype was the most common in all collections, with AfrCa2c usually the next most frequent (Nu12, Nu15, Hi06, Hi08, Hi11, Fig 4A). An additional nine minor haplotypes were found that were far more sporadic in their distribution. A similar set of profiles were observed in the two migratory destinations surveyed in Nebraska (NE14) and Pennsylvania (PA15), which again showed a AfrCa1c majority and a sizeable AfrCa2c contribution. Three additional minor haplotypes were observed in the Nebraska collection, two of which were also found in Texas. The one exception, w01, was only represented by a single specimen.

A, shows the location of the collection sites (red stars) as described in Fig 2. Pie charts show relative proportions of the thirteen TpiI4a200 haplotypes from each collection. In brackets under the year are the number of specimens analyzed. B, TpiI4a200 haplotype profile from the pooled Texas collections (n =  129). C, TpiI4a200 haplotype profile from the pooled Mexico collections (n =  134).

The Mexico collections displayed greater variation in their TpiI4a200 profiles. The AfrCa1c haplotype that was the majority in Texas was also predominant in eight of the 13 Mexico collections but was not observed in the remaining four collections (Du13, Du14, Ta13, Ta14, Fig 4A). The AfrCa2c haplotype found in all U.S. collections was not found in six Mexico collections yet was the majority haplotype in Chiapas in 2016 (Ch16) and Durango in 2013 (Du13). The AfrCa1a haplotype was a minor component in U.S. FAW populations but was the sole haplotype found in Tamaulipas (Ta13, Ta14). As with the CSh haplotypes, the TpiI4a200 profile changed substantially in Durango and Chiapas over time, with the most recent findings for each (Du16 and Ch16) differing from earlier collections.

Pooling of the Texas collections showed the general predominance of AfrCa1c and identified five TpiI4a200 haplotypes (w05, w07, w08, w10, and w12) that were not found in the Mexico collections (Fig 4B). The pooled Mexico data showed that despite differences between collections the overall distribution of haplotypes was like that observed in Texas, with AfrCa1c generally the most common variant found and five of the six minor haplotypes a subset of those detected in Texas (Fig 4C). The one exception, AfrCa2b, was present in three Mexican collections where it was a major component of FAW populations in Durango in 2013-2014 (Du13, Du14), and a minor constituent of Chiapas in 2013 (Ch13).

### Climate and other environmental factors

**Fig 5:**
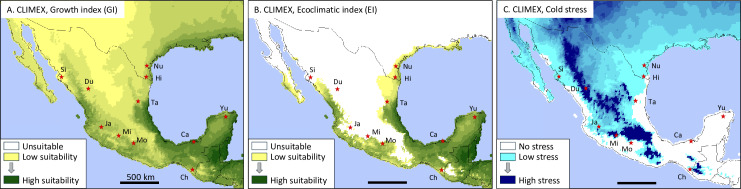
CLIMEX modeling analysis of surveyed region. Maps derived from CLIMEX modeling. A, Growth index based on irrigation scenario and FAW parameters from Timilsena et al (2022) [2]. B, Ecoclimatic index using same set of parameters. C, map of Cold stress using same parameters.

The climate suitability program, CLIMEX, was used to identify where FAW populations could be routinely supported in Mexico based on climate suitability parameters. The annual Growth Index (GI) describes the potential for FAW growth based on periods of favorable temperature and moisture conditions, with higher values indicating more suitable conditions. While all of Mexico has the potential for supporting FAW development at certain times of the year, for most of northern and central Mexico the conditions lead to marginal GI values even with assumptions of irrigation during the agricultural seasons (Fig 5A). Integrating the annual GI (with irrigation) with estimates of where and when temperature and moisture are unfavorable to FAW development gives the Ecoclimatic Index (EI), which projects the likelihood of permanent or year-round FAW populations. These are of interest because of their potential to serve as migratory sources for less suitable locations. Based on this analysis, permanent populations in Mexico are limited to coastal and the most southern states (Fig 5B). Of the collection sites in this study, those in Sinaloa (Si), Durango (Du), and Jalisco (Ja) appear least capable of consistently supporting permanent FAW populations, suggesting that infestations in these locations may originate from outside sources.

CLIMEX modeling of temperatures unsuitable for FAW development indicates that cold stress is the most prominent environmental factor limiting permanent populations (Fig 5C). Most of central Mexico, the northern Mexican states, and the United States show significant cold stress, which in Mexico corresponds to regions of higher elevation (compare Fig 5C with Fig 2 and 4). Areas of no cold stress correspond to locations with high EI suitability.

**Fig 6:**
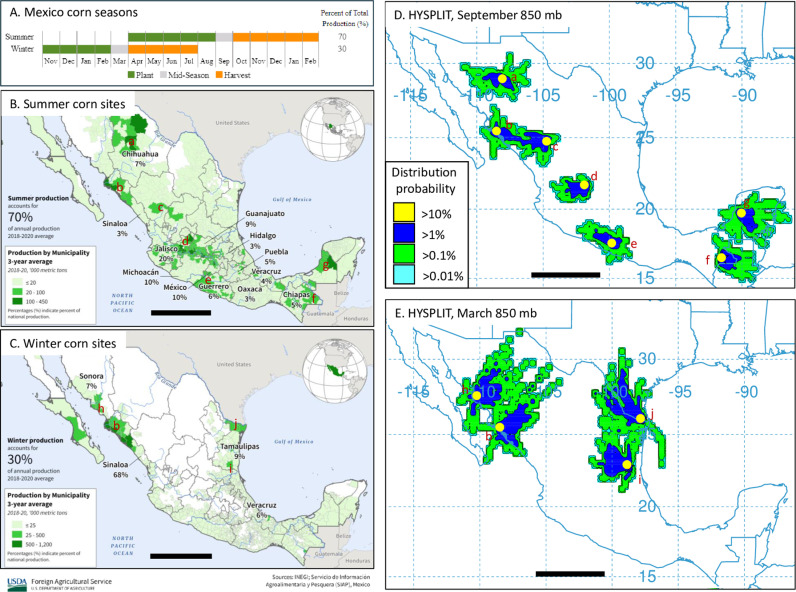
Distribution of major corn production sites in Mexico and projected single night dispersion pattern during the summer and winter mid-season months. A-C are public data from INEGI; Servicio de Infromación Agroalimentaria **y** Pesquera (SIAP), Mexico and USDA-FAS. A, timing of the Mexican summer and winter corn growing seasons. B, locations of major corn production in Mexico during the summer growing season. C, locations of major corn production in Mexico during the winter growing season. D, HYSPLIT forward projections of 12-h overnight trajectories based on average wind data for the summer mid-season month of September. E, maps derived from HYSPLIT forward projections of 12-h overnight trajectories based on average wind data for the winter mid-season month of March. Site coordinates: a, Chihuahua (28.88, -106.55); b, Sinaloa (25.39, -108.44); c, Durango (24.14, -104.14); d, Jalisco (20.33, -102.68); e, Guerrero (18.33, -100.49); f, Chiapas (16.59, -91.46); g, Campeche (20.05, -90.39); h, Sonora (27.83, -109.66); i, Tamaulipas (22.67, -98.93); j, Hidalgo TX (26.09, -98.26).

To further estimate regions capable of supporting high-density FAW C-strain populations the locations of the major corn producing areas were identified. There are two corn growing seasons in Mexico, with 70% of production resulting from plantings in the spring (summer corn) and the remainder from winter plantings for the winter corn season (Fig 6A). Summer corn production occurs through much of Mexico, from the northern state of Chihuahua to Chiapas and Campeche in the south (Fig 6B). Winter corn production is more restricted with about 80% of corn production during this period coming from locations in the states of Sonora, Sinaloa, and Tamaulipas (Fig 6C).

The HYSPLIT program was used to estimate the degree to which seasonal wind patterns might direct FAW movements during the two growing seasons from these high corn production locations. Projections were made based on wind patterns during the midseason months, September for summer corn, March for winter corn (Fig 6A). The HYSPLIT modeling for summer corn showed mostly localized dispersion with little evidence of strong or consistent directionality (Fig 6D). Projections made for March, the mid-season of the winter corn season showed greater directionality from the high corn production sites, which are mostly limited to coastal states in northern Mexico. Projections from Hidalgo County, TX (j) shows a substantial northward bias, consistent with FAW from southern Texas migrating en masse into the central United States during the early spring [3,33], while projections from Tamaulipas (i) indicates that interactions between FAW from this location and Hidalgo County are plausible. HYSPLIT projections from Sonora (h) and Sinaloa (b) show a strong northeastern trend, suggesting a possible contribution to western Texas and Arizona. However, this possibility is problematic because of the absence of high corn acreages along the dispersal pathway (compare Fig 6C and 6E).

## Discussion

The distribution of FAW in the United States is dominated by the annual northward movement of populations from overwintering locations in southern Florida and the Mexico-Texas border regions [3,33]. This long-distance dispersal behavior is consistent over time based on population-specific haplotype profiles that show reproducible patterns at the migratory destinations over different years [11,18,33]. In addition, there is typically little genetic differentiation between the source and destination populations despite the migration occurring over multiple generations and extending over thousands of kilometers. This result is consistent with the movements of large FAW populations, made possible by the northward progression of high-density corn plantings during the spring-summer growing season and facilitated by seasonal wind patterns that show consistent year-to-year velocity and directionality [3,4]. While CLIMEX projections indicate that isolated overwintering groups are possible along the more temperate locations on the Gulf Coast outside of the primary overwintering areas (Fig 5AB), their contributions to the genetic composition of the migratory wave will be small compared to the massive migratory populations expected from southern Texas and Florida.

This was demonstrated with the data from Texas, where the eight collections from five locations and spanning seven years (2004-2011) show similar COIB haplotype profiles characterized by the predominance of CSh2 and roughly equal proportions of CSh1 and CSh4 (Fig 2A). Similarly, the five Texas collections showed the same TpiI4a200 pattern of a predominant AfrCa1c and a significant AfrCa2c contribution, as did the two migratory destinations of the Texas populations at Scottsbluff Co, NE and Erie Co, PA that lie thousands of kilometers to the north. This is the pattern to be expected from the mass migration of a high-density population, which will tend to preserve the haplotype profile of the source population at the migratory destinations.

In contrast, the temporal and geographic distribution of genetic markers in Mexico indicate a different dynamic dictating the distribution of FAW in that country. Distinct haplotype profiles were observed in the same location for different years (CSh haplotypes in Du14-16, Fig 2A) and between locations in the same year (TpiI4a200 in Du14, Mi14, and Ta14, Fig 4A). Of particular interest is the observation that both colony and field collections from southern Tamaulipas (Ta13 and Ta14) had both CSh and TpiI4a200 haplotype profiles markedly different from that observed in most of the other collections (Fig 2A and Fig 4A). Large genetic differences were also observed between the 2013 collections (Si13, Du13, Ch13, and Ta13) but these should be viewed with some reservation as they are derived from laboratory colonies that at best can only approximate the composition of the field populations they represent. In two cases, Du14 and Ta14, the colony results were supported by field surveys performed the following year, but similar confirmation was not done for Si13 or Ch13. Despite these caveats, we believe the overall genetic data indicate limited and inconsistent interactions between FAW from different locations that can produce transient genetic differentiation between collections.

We conclude that unlike in North America, the FAW in Mexico appear to be limited to local dispersal behavior defined by climatic, geographical, and agricultural factors. Climatic conditions that can support year-round FAW populations are mostly limited to the eastern and western coastal states, in between which lies the Chihuahua Desert highlands that are characterized by high cold stress for FAW (Fig 5C). Mean seasonal wind patterns for Mexico show low velocity and inconsistent directionality all year [16], and so are not favorable for long-distance transport, particularly across the central desert region where host availability is often low (Fig 6C). This would tend to isolate the FAW populations on the eastern and western coasts. In addition, while FAW is known to have a broad host range, the mass migration observed in the United States is dependent on the extensive corn agriculture that provides an ample and contiguous supply of host plants for the multigenerational northward progression of high density populations [3,4]. In comparison, the high corn production locations in Mexico for both growing seasons are geographically dispersed rather than continuous (Fig 6B,6C). These potentially create “islands” of high-density FAW that in the absence of strong seasonal winds may not consistently exhibit long-distance flight and thereby may only have sporadic interactions with each other (Fig 6D).

We postulate that these conditions generate the transient examples of genetic structure observed by our haplotype methodology. Specifically, the weak seasonal winds with variable directionality could lead to annual variability in the source populations of the FAW infesting locations unable to support permanent populations. The latter is a likely explanation for the observed genetic variability in the Durango collections from different years, given the unfavorable EI Index for the location (Du, Fig 5B). And while the Chiapas collections are from locations with more favorable EI Index metrics, the relatively low corn availability during the winter season (Fig 6C) could reduce local population size and thereby increase the genetic impact of outside introductions.

These conjectures were further examined using HYSPLIT modeling, which projects dispersion patterns based on wind activity. During the summer corn mid-season month of September HYSPLIT projections generally show limited wind-dependent dispersal from the major corn producing locations, consistent with these populations being relatively isolated. While longer distance dispersal is projected during winter corn mid-season month of March, the small number of high production corn sites during this period and the projected dispersal from those sites suggest that substantial and consistent interactions between populations on the eastern and western coasts are unlikely. Specifically, we see no indications that mean seasonal wind patterns during periods of high corn production are strong enough to direct a large migratory population across the Chihuahua Desert. Taken together, these findings indicate that migration on the scale observed in the United States is unlikely for the FAW populations in Mexico.

**Fig 7:**
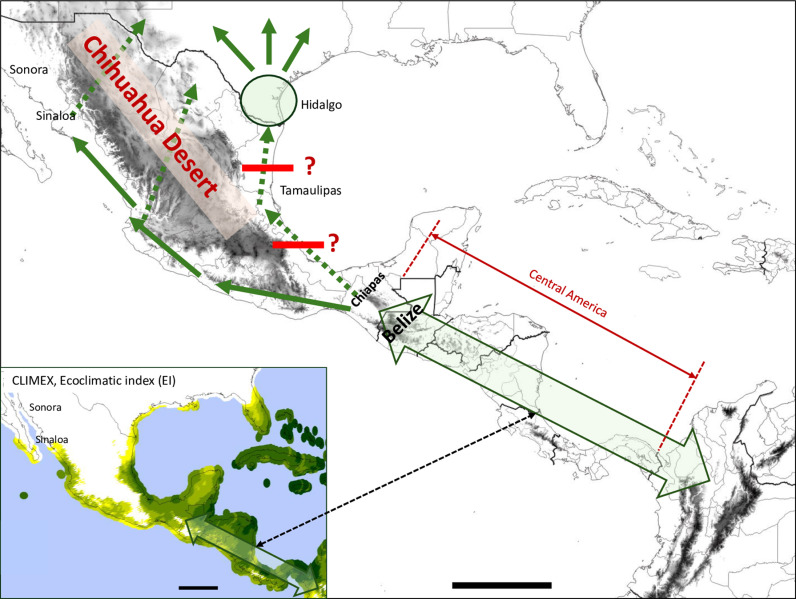
Projected northward movement of FAW through Mexico. Topographical map (QGIS) showing the possible movements of FAW through Mexico and their potential interaction with Texas populations in Hidalgo County. Inset shows CLIMEX EI index map indicating high climate suitability in Central America for FAW (double block arrow). Solid arrows indicate likely migration pathways. Dotted arrows indicate movements that appear to be limited or sporadic based on environmental barriers or the distribution of genetic markers.

The implications to FAW movements across the Mexico-Central America land corridor is summarized in Fig 7. While little is currently known about the distribution of FAW populations in Central America, CLIMEX analysis indicates that the region from northern South America to Belize has the potential to support a panmictic population through Central America into southern Mexico (block arrow, Fig 7). After the Mexican state of Chiapas, it appears that the Chihuahua Desert forces a bifurcation of any further northward movement to either the western or eastern Mexican coasts. The western FAW continues to be separated from those on the eastern coast by desert highlands as far north as Sonora, after which conditions become less suitable for FAW development. These conditions make it unlikely that the FAW from the winter corn production areas in Sonora and Sinaloa can consistently interact with populations in Texas (Fig 7).

Limitations in the movement of FAW along the eastern coast are suggested by the Tamaulipas collections that in 2013 and 2014 had markedly different haplotype profiles from those seen in Hidalgo County to the north or Chiapas to the south (Fig 4A). Additional sampling is needed to test the persistence of this pattern. The available data indicate that the movement of FAW in this region is not sufficient to consistently homogenize populations with respect to the relative frequencies of the major tested haplotypes (Fig 7). This could result from weak wind vectors and the scarcity of high production corn locations along the eastern coast. While this conclusion is preliminary in lieu of additional confirmatory surveys it is consistent with the absence of those conditions that support long-distance mass migration in the United States.

In conclusion, the occasional and transient genetic structure in Mexico discovered using haplotype frequency comparisons are consistent with climate suitability and air transport projections that support localized dispersal behavior over that of long-distance migration. The results indicate that the exchange of FAW between the two American continents through Central America and Mexico does not involve migratory movements of mass populations as observed in the United States.
